# Nanoparticle ultrasonication: a promising approach for reducing bacterial biofilm in total joint infection—an in vivo rat model investigation

**DOI:** 10.1186/s42836-024-00279-7

**Published:** 2024-11-06

**Authors:** Itay Ashkenazi, Mark Longwell, Barbara Byers, Rachael Kreft, Roi Ramot, Muhammad A. Haider, Yair Ramot, Ran Schwarzkopf

**Affiliations:** 1 Department of Orthopaedic Surgery, NYU Langone Health, New York, NY 10003 USA; 2 Division of Orthopaedic Surgery, Tel-Aviv Sourasky Medical Center, 6329302 Tel-Aviv, Israel; 3 Center for Excellence in Biofilm Research, Allegheny Health Network Research Institute, Pittsburgh, PA 15224 USA; 4 Ramot Biomedical Eng. Ltd., Maas, 4992500 Israel; 5 Dimoveo Medical, Or Yehuda, 6037606 Israel

**Keywords:** Periprosthetic joint infection (PJI), Ultrasonication, Biofilm, Animal model, DAIR

## Abstract

**Background:**

While the benefits of sonication for improving periprosthetic joint infection (PJI) are well-documented, its potential therapeutic effect against bacterial biofilm remains unstudied. This study aimed to investigate the safety and efficacy of a novel nanoparticle ultrasonication process on methicillin-resistant *Staphylococcus aureus* (MRSA) bacterial biofilm formation in a PJI rat model.

**Methods:**

This novel ultrasonication process was designed to remove attached bacterial biofilm from implant and peri-articular tissues, without damaging native tissues or compromising implant integrity. Twenty-five adult Sprague–Dawley rats underwent a surgical procedure and were colonized with intra-articular MRSA, followed by the insertion of a titanium screw. Three weeks after the index surgery, the animals received a second procedure during which the screws were explanted, and soft tissue was sampled. The intraoperative use of the nanoparticle sonication treatment was employed to assess the device’s safety, while ex vivo treatment on the retrieved tissue and implants was used to evaluate its efficacy.

**Results:**

Clinical and histological assessments did not indicate any macro- or micro-damage to the host tissue. Sonication of the retrieved tissues demonstrated an average bacterial removal of 2 × 10^3^ CFU/mL and 1 × 10^4^ CFU/gram of tissue. Compared to the standard-of-care group (*n* = 10), implants treated with sonication (*n* = 15) had significantly lower remaining bacteria, as indicated by crystal violet absorbance measurements (*P* = 0.012).

**Conclusions:**

This study suggests that nanoparticle sonication technology can successfully remove attached bacterial biofilms from explanted orthopedic hardware and the joint capsule, without negatively affecting native tissue. The study provides initial results supporting the potential of nanoparticle sonication as an adjuvant treatment option during a DAIR (debridement, antibiotics, and implant retention) procedure for PJI, paving the way for future clinical trials.

## Background

The incidence of periprosthetic joint infection (PJI) following primary total knee arthroplasty (TKA) has been reportedly between 1–2% [1]. While not considered a common complication, PJI can result in increased patient morbidity and mortality [2, 3], as well as increasing healthcare costs. Recent evidence showed that the economic burden of PJIs in the United States was estimated to reach $1.85 billion by 2030 [4], underlining the importance of efficient PJI management.

Understanding the microbiological spectrum and pathogenesis of PJIs is crucial for clinical management. *Staphylococcus aureus* and coagulase-negative *staphylococci* are the most common pathogens in acute and chronic PJI respectively, accounting for approximately half of all PJI cases [5]. These pathogens form biofilms [6], which complicates the treatment of PJI. It is generally accepted that mature biofilm develops within four to six weeks [7]. Biofilm production includes adherence of bacteria to implants, multilayer cellular proliferation, and cell-to-cell adhesion, resulting in complex 3D-bacterial communities [7]. The biofilm allows microorganisms to enter a stationary state, increasing their resistance to antimicrobial agents, causing a persistent, hard-to-treat infection [7]. In the setting of PJI following TKA, the presence of bacterial biofilms usually necessitates explantation of the infected implant, in either a one- or two-stage revision procedure [8].

In contrast, in the dental literature, sonication has been extensively studied as a therapeutic method for treating biofilms and its efficacy has been documented in relation to soft tissue infections and the promotion of wound healing [9, 10]. While the utilization of low-intensity sonication to disrupt biofilm on extracted implants for diagnostic purposes has demonstrated higher sensitivity compared to conventional periprosthetic tissue cultures [11, 12], its utility as a treatment modality in a PJI biofilm model has yet to be investigated.

A novel nanoparticle ultra-sonication process (Dimoveo Medical, Israel) was developed to address PJI biofilm. This process was designed to remove attached bacteria biofilm from the implant and peri-articular tissues, such as the joint capsule, without damaging native tissue or compromising implant integrity. Previous in vitro pilot work evaluated the aforementioned process against biofilm formation on titanium screws and coupons [13]. The above pilot in vitro studies demonstrated the efficacy of the technology against methicillin-resistant *Staphylococcus aureus* (MRSA) biofilms. Therefore, in this study, we investigated this novel nanoparticle ultra-sonication process on methicillin-resistant *Staphylococcus aureus* bacterial attachment and biofilm formation in a periprosthetic joint infection in a rat model. We hypothesize that the novel nanoparticle sonication technology can remove attached bacteria biofilm from orthopedic hardware and the joint capsule without causing damage to native tissues.

## Methods

### Study design

**Fig. 1:**
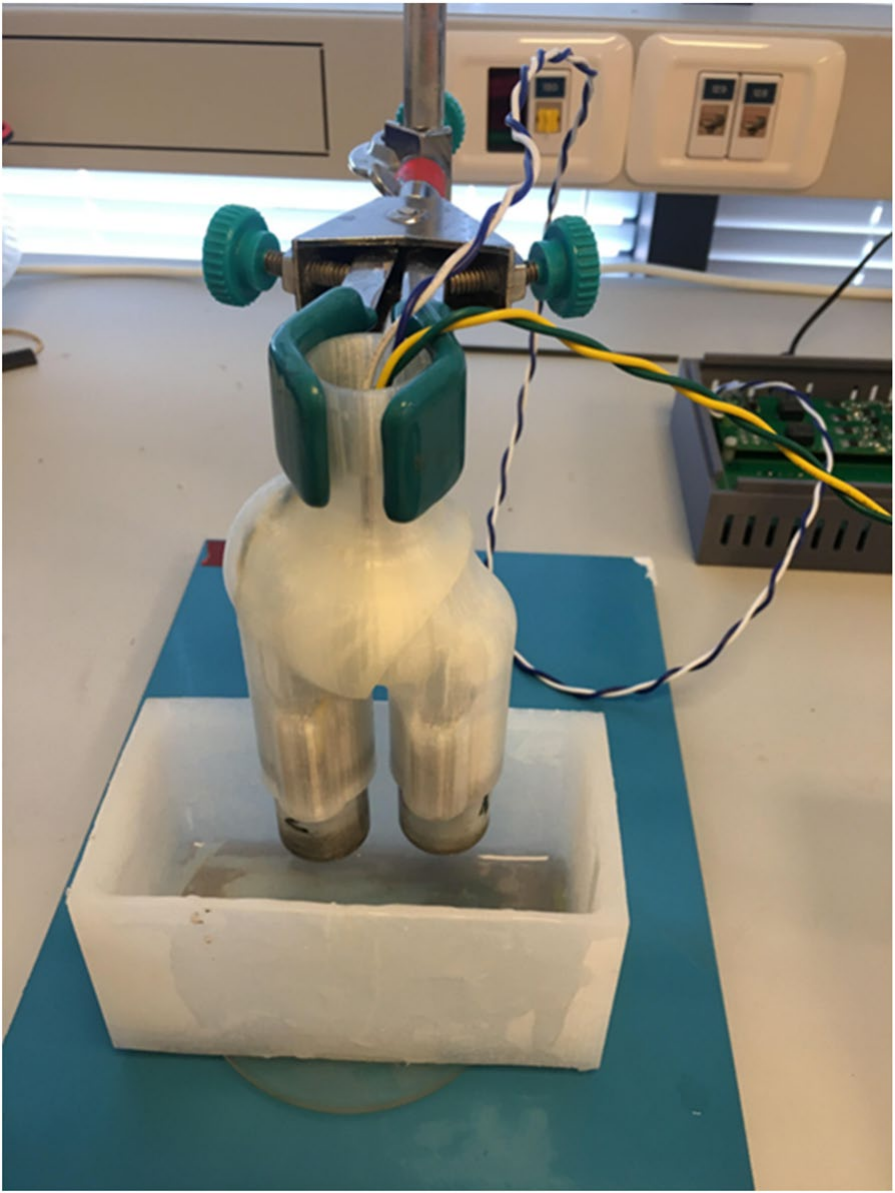
Nanoparticle sonication construct

This was an animal-model study on 25 adult male Sprague–Dawley rats (about 350 g). All of the participating animals underwent a surgical procedure during which they were injected with a methicillin-resistant *S. aureus* solution into a pre-drilled intra-articular tibial canal, followed by the insertion of a titanium screw. On postoperative day (POD) 21, a second procedure was done and included the explanation of the titanium screw as well as the collection of the joint capsule and periarticular tissue specimens. The nanoparticle sonication treatment was intraoperatively employed to assess the device’s safety, while the retrieved tissue and implants were treated ex vivo to evaluate its efficacy. Due to the small size of the animal models’ joints, in vivo testing of the nanoparticle construct was not possible for the present investigation. The nanoparticles utilized were made of iron oxide. Figure 1 presents an example of the nanoparticle setup, consisting of the sonication construct and silicone container. During each nanoparticle treatment, only the affected leg of the rat was put in the silicone container. The container was sterilized between treatments to prevent contamination.

The justifications for choosing a rat model for simulating periprosthetic joint infection were based on a few factors. First, the proposed simulated arthroplasty implant materials could be obtained for the animal model [14]. Second, the novel nanoparticle sonication system was designed to meet the size requirements of the animals. Lastly, the PJI rat model had been previously established with the ability to monitor the course of the acute and chronic phases of infection [15], and a time point of 21 days following infection induction has shown to have a robust and stable biofilm on titanium screws [14].

### Animal study ethical considerations

This study was approved by the Institutional Animal Care and Use Committee (IACUC) of the Allegheny-Singer Research Institute (ASRI). Twenty-five adult male Sprague–Dawley rats (roughly 350 g) were obtained and given facility chow and water ad libitum. Twenty-five rats were utilized after conducting a power calculation using a pre-set alpha of 0.05 and a power of 95%. After accounting for the mean crystal violet readings for our treatment (0.4) and SOC (1) groups, we calculated that we would require 14 and 9 animals in the treatment and SOC groups, respectively. Tables 1 and 2 detail our parameters and calculations. Animals were housed at the ASRI animal care facility with a fully-equipped operating room. Animals were housed in solid bottom cages with soft bedding and enrichment. Animals were housed in pairs whenever possible to provide companionship and limit environmental stress. Animals were acclimated for a minimum of 3 days preoperatively. After surgery and inoculation, animals were housed under BSL-2 conditions.

**Table 1:** Sample size calculation parameters

Study parameters	
Mean, treatment group	0.4
Mean, SOC group	1
Alpha	0.05
Beta	0.05
Power	0.95

**Table 2:** Sample size calculation

Sample size	
Treatment group	14
SOC group	9
Total	23

### Surgical procedures and perioperative management

Surgical procedures were performed as previously described by Fan et al. [16] and by Harrasser et al. [17]. On the day before surgery, preoperative baseline measurements were performed, including weight and local temperatures. Buprenorphine at 0.5 mg/kg IP was administered 30 min before surgery. Rats were anesthetized with 1%–3% isoflurane in 1 L of O_2_/min. A 3–4 cm skin incision was created on the superior-lateral aspect of the surface of the right femur from the supracondylar region to the tibia of the right lower limb. An intercondylar canal was drilled in the tibia and 2.5 μL containing 1 × 10^6^ MRSA was injected, after which a titanium maxillofacial screw (1.5 mm in diameter, 5 mm in length) was implanted. The nanoparticle and SOC animals received the same surgical procedure. The wound was closed using 5–0 Vicryl deep dermal sutures. Postoperative rats received one dose of Buprenorphine-SR, 5 mL of fluids, and meloxicam (a non-steroidal anti-inflammatory drug). No postoperative antibiotic treatment was administered to either group. At 24 and 48 h postoperatively, animals received an additional dose of meloxicam to encourage weight-bearing and mobility.

On POD 21, animals of both groups were anesthetized as previously described. The tibial screws were removed aseptically for further treatment and analysis. Additionally, joint capsule swabs, as well as soft tissue and bone specimens, were collected from the infected knee. Sequentially, the native knee was surgically opened, as previously described, and subjected to treatment via the novel system to assess the *in-vivo* safety of the nanoparticle sonication device. Each animal was placed in a silicone container. The container was filled with 300 mL of Hanks’ Balanced Salt Solution (HBSS) with 12 mg of nanoparticles. The sonication device was applied directly above the surgical incision for 15 min. Upon completion of the treatment, the animal was euthanized by CO_2_ inhalation. The surgical incision was not sutured. A cardiac puncture was performed to collect systemic blood for future measurements.

### Outcome measures

Upon completion of the animal study, the following tests were performed to assess the safety and efficacy of the novel system.

Tests to assess the safety of the novel device (Dimoveo Medical, Israel) were performed on the study group only (nanoparticle procedure). These tests included monitoring of the vital signs during the nanoparticle sonication process to identify any signs of pain or distress, as well as clinical images and histopathological assessment of bone and soft tissues. The clinical and histopathological assessments were done on the left femur of the animal, where no infection occurred, to ensure that there were no negative effects of the infection on safety performance. Clinical assessment included pre- and post-sonication clinical images of the joint to identify any signs of gross damage to the tissues. For histological assessment, tissue and bone were fixed in paraformaldehyde and paraffin-embedded. Serial sections of the material were created. A board-certified clinical pathologist reviewed all sections via an EVOS FL at 20X magnification to identify any signs of local damage to the soft tissue or bony elements that were in contact with the screw (These included samples from the tibia, femur, cartilage, and ligaments within the knee). Histological evaluation was performed immediately after sonication to ensure no mechanical damage had occurred. As the experimental animals were euthanized shortly following testing, for regulatory purposes, the long-term effects of nanoparticle sonication could not be assessed in the present study.

Tests to assess the efficacy of the novel device were performed on both the study and the control groups and included the following: (1) *Joint Capsule Assessment:* A swab sample from the joint capsule was used for colony-forming units (CFU) calculations. The samples were submerged in Hanks' Balanced Salt Solution (HBSS) in a 15-mL polystyrene conical Falcon centrifuge tube. To detach bacteria from the sample, vortexing and sonication were used. Samples were serially diluted and plated onto Brain Heart Infusion (BHI) plates. After 24-h incubation at 37 ºC, the CFU were counted for the appropriate dilution and CFUs/mL were calculated. (2) *Explanted Tissue Assessment:* To calculate bacteria removed using the novel technology, the tissue sample was weighed and then placed in a silicon container and 25 mL of HBSS was added containing 12 mg/300 mL of nanoparticles. All 25 tissue samples were sonicated for 15 min, using the novel device. At the end of the sonication, the magnetic beads were removed from the solution. Samples were then serially diluted and plated onto BHI plates. After 24-h incubation at 37 ºC, the CFU was counted for the appropriate dilution, and CFUs/mg of tissue were calculated. (3) *Explanted Screw Assessment*: The explanted screws were subjected to SOC or nanoparticle treatment. For the SOC group, following a 50-mL lavage step, the screw was placed into a sterile polystyrene centrifuge tube containing 1 mL of HBSS. The sample underwent 3 cycles of vortexing for a period of 10 s to remove bacteria. The samples were then serially diluted and plated onto BHI plates. After a 24-h incubation at 37 ºC, the CFUs were counted for the appropriate dilution, and CFUs calculated. For the nanoparticle treatment group, the screw was placed in the silicon container and 20 ml of HBSS was added containing 12 mg/300 mL of nanoparticles. A volume of 20 mL was utilized as it represents the minimum volume of solution necessary to operate the transducer. The sample was sonicated for 15 min with two ultrasound transducers set at 40 kHz and at 2.5 watts per square centimeter. The transducers were in direct contact with the nanoparticle-enriched solution and not in contact with the screws or tissue. At the end of sonication, the magnetic beads were removed from the solution. Samples were then serially diluted and plated onto BHI plates. After 24-h incubation at 37 ºC, the CFUs were counted for the appropriate dilution, and CFUs were calculated. Screws of both groups were retained for crystal violet analysis. Previous studies have demonstrated the efficacy of sonication with nanoparticles over sonication alone [13]. Sonication without nanoparticles has been shown to take considerable time and was not utilized as a control group for the present analysis. (4) *Crystal Violet.* The explanted screws were rinsed twice in wells with HBSS to remove non-adherent bacterial cells. The screws were covered with a crystal violet solution at room temperature for 15 min. The screws were rinsed with distilled water until the rinsing solution was clear and completely dried. Once dried, the crystal violet was solubilized with 97% ethanol for 10 min. Following solubilization, the ethanol solution was moved to a clean well, and a spec reading was taken at 600 nm.

### Data analyses

Data were organized and collected using Microsoft Excel software (Microsoft Corporation, Richmond, Washington, USA). The data collected were described as average and median for continuous variables. All data analyses were performed by using the Microsoft Excel statistical tool (Microsoft Corporation, Richmond, Washington, USA). For continuous variables, the Mann–Whitney Score was used to compare differences between the study and the control groups. A *P*-value of less than 0.05 was considered statistically significant.

## Results

### Animal model

All 25 animals made it to the study end date. While some animals lost weight initially, all had gained weight by 21 days with an average weight gain of 77 g. All animals at 21 days after the index procedure showed various signs of inflammation, including redness, tenderness, and increased temperature at the implanted joint. When the joint capsule was opened, the surgeon visually observed that 21 of 25 animals had a cloudy serous fluid within the space and evidence of scar tissue within the knee joint. Bone loss was apparent in 17 of 25 animals, as evidenced by loose screws at the time of euthanasia.

### Nanoparticle treatment safety

**Fig. 2:**
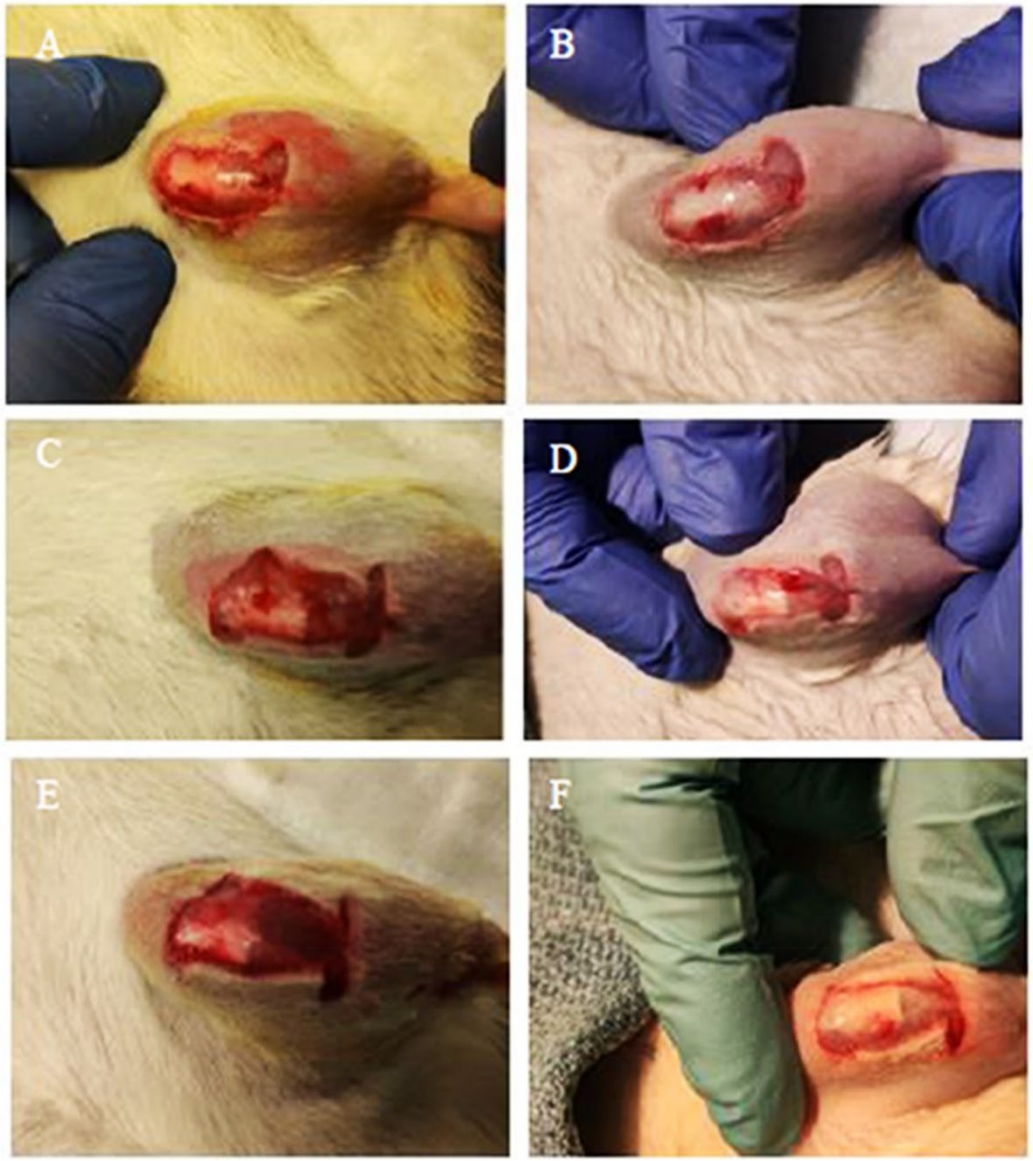
Clinical images of the joint capsule of the left rear rat leg of three individual animals. **A**, **C**, and **E** were taken prior to the nanoparticle treatment while **B**, **D**, and **F**, respectively, were taken following the nanoparticle treatment. The pre- and post-treatment images showed that the nanoparticle sonication device did not cause any visual damage

**Fig. 3:**
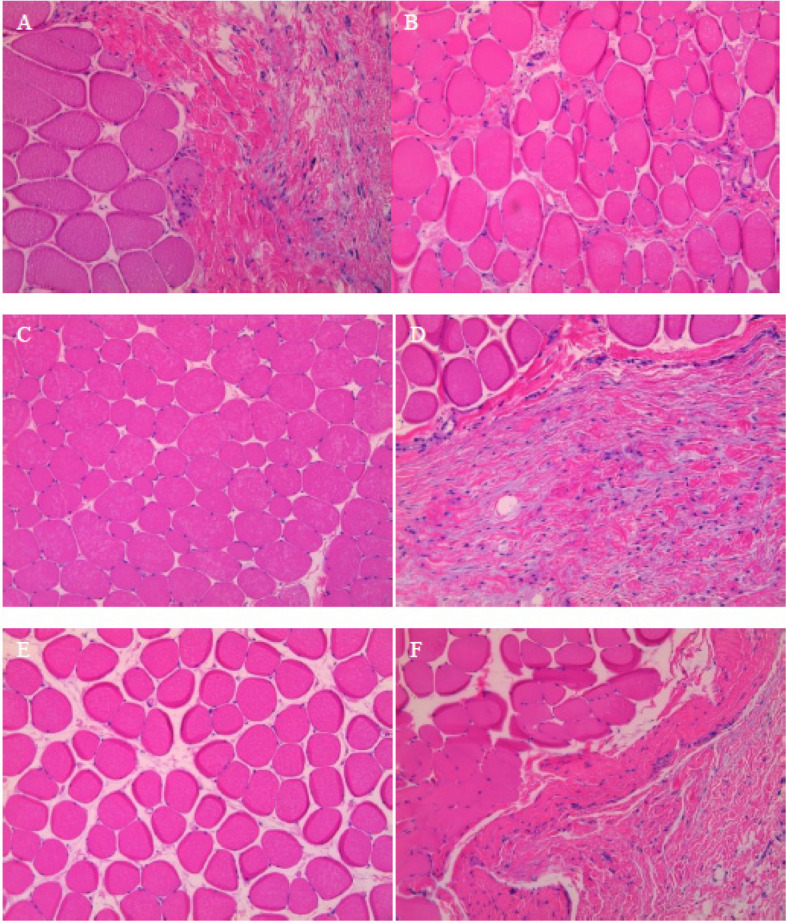
Histopathological examination of the joint tissue following nanoparticle sonication of six individual study animals (**A** to **F**), exhibiting intact cellular structures with no evidence of remaining nanoparticles

Animals tolerated sonication under anesthesia without any reported signs of pain, distress, or adverse events. The visual inspection before and after sonication with the nanoparticle device showed no damage to the joint capsule in all the animals (Fig. 2). Similarly, in all the animals, the histopathological examination of the nanoparticle-sonicated tissue did not indicate any damage to the host tissue (Fig. 3). Furthermore, histopathological examination revealed no nanoparticles present within the host tissue, suggesting that the particles were washed away with lavage. Additionally, the liquid provided a medium for the ultrasonication to work effectively.

### Nanoparticle treatment efficacy

The joint capsule swab collected at 21 days showed evidence of MRSA growth in all 25 study animals. A total of 25 tissue samples underwent nanoparticle treatment to calculate the nanoparticle removal of bacteria from the tissue. Results from 25 individual tissues demonstrated an average bacterial removal of 2 × 10^3^ CFU/ml and 1 × 10^4^ CFU/gram of tissue.

**Fig. 4:**
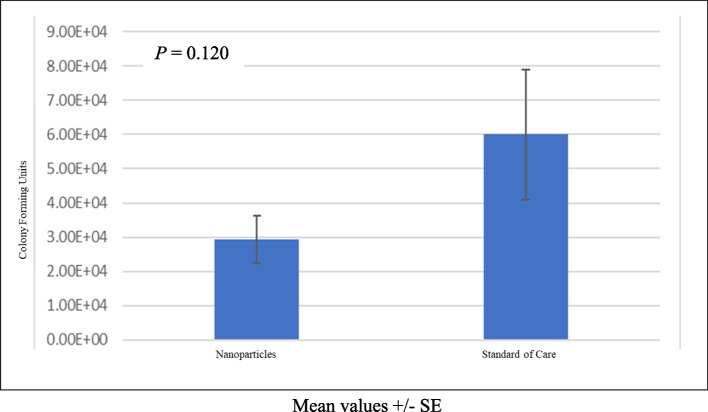
Bacteria remaining after treatment. A total of 25 screws were analyzed. Mean screw colony-forming units (CFU) after treatment were compared between the nanoparticle treatment group (*n* = 15) and the standard-of-care (SOC) treatment group (*n* = 10). Error bars represent standard error (SE)

**Fig. 5:**
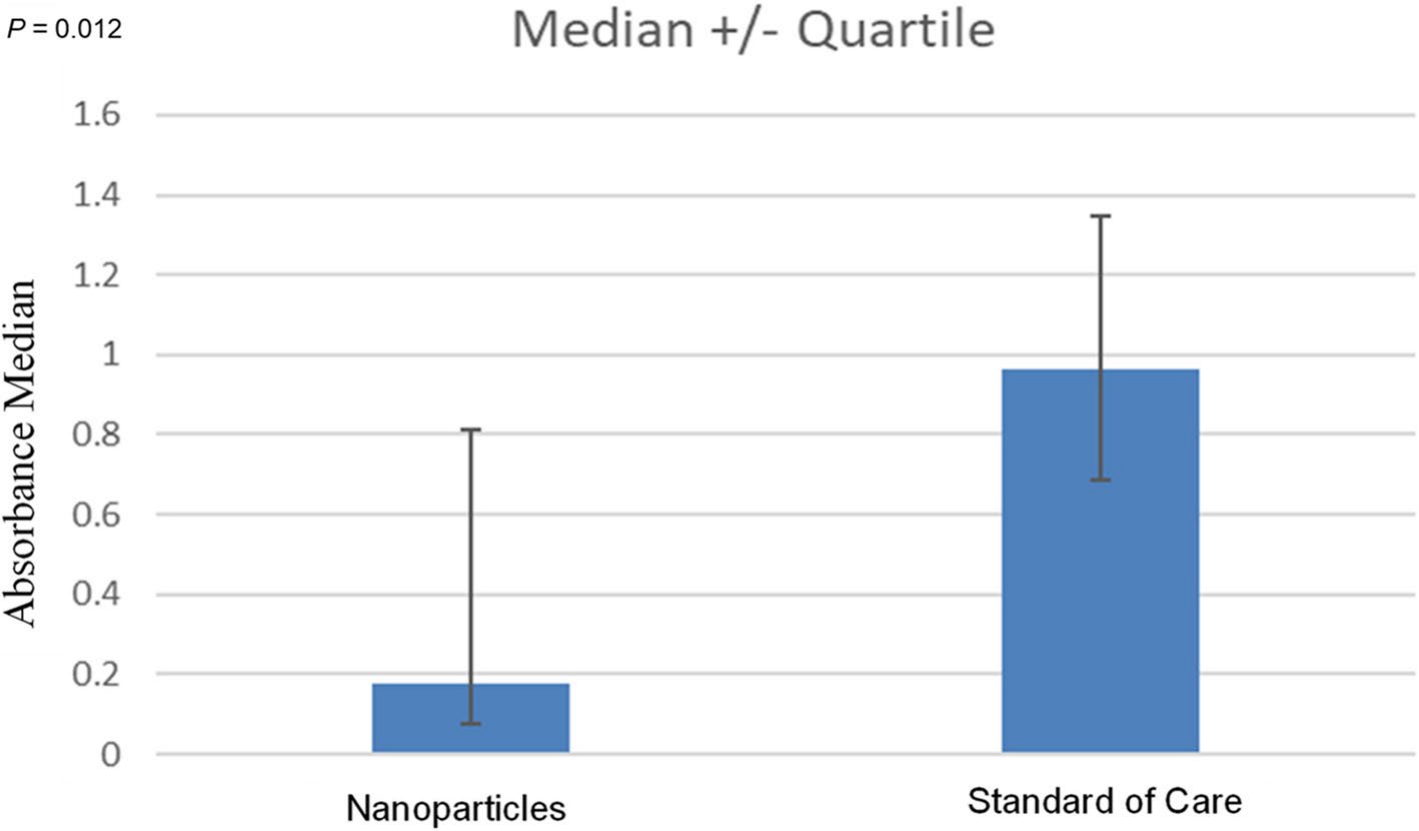
Crystal violet (CV) staining of screws. The nanoparticle bar represents the absorbance median of the crystal violet results for the 15 screws assigned to the nanoparticle treatment. The standard-of-care (SOC) bar represents the median of the crystal violet results for the 10 screws assigned to the SOC treatment

When evaluating the bacteria remaining on the screws after nanoparticle sonication against SOC treatment, the median bacteria remaining after SOC treatment was over fivefold higher than after the nanoparticle treatment, although this was not statistically significant (3.22 × 10^4^ vs. 6.20 × 10^3^, respectively, *P* = 0.120) (Fig. 4). Correspondingly, the median crystal violet absorbance in the SOC group was more than fivefold higher than the median absorbance reading of the nanoparticle group, indicating a significantly higher number of remaining bacteria (0.96 vs. 0.17, respectively, *P* = 0.012) (Fig. 5). As evidence of MRSA growth was seen via the joint capsule swab, bacteria removed from the implant were not cultured. Furthermore, this might represent another instance where the nanoparticles demonstrate superiority over the SOC treatment.

## Discussion

This animal-model study was the first study to assess, in vivo, the safety and efficacy of a novel nanoparticle sonication device in treating bacterial biofilm on peri-articular knee tissue and simulated arthroplasty implant materials. The primary findings of the study were as follows: (1) At 21 days, all animals had recovered bacteria from the inoculated joint, supporting the validity of the animal model used for this study; (2) the use of the novel nanoparticle sonication device was safe, as indicated by both clinical and histological assessments of the soft tissue; (3) the nanoparticle device removed a substantial portion of bacteria from the tissue; and (4) when compared to the standard-of-care treatment, implants treated by the nanoparticle sonication device had significantly less remaining bacteria, as assessed by both a direct measurement and crystal violet absorbance.

The findings of our study suggest that all the animals in the study had an active infection at the time of screw explanation, and all study screws were coated in bacterial biofilms at the time of explanation. This is in accordance with a previous study by Glage et al., who reported the formation of a robust biofilm on explanted titanium screws 21 days after infection induction with *S. aureus* injection into bone drill holes [14]. This further helps validate that the animal model utilized in the study produced both a periarticular infection and implant biofilm formation, further supporting and validating the findings of this study. Earlier time points were not assessed in this study, as the goal was primarily to evaluate the efficacy of the nanoparticle technology and not the bacterial load at different time points. This may be evaluated in future studies focusing on biofilm generation in animal models.

This study further validated previous in vitro studies that demonstrated the efficacy of the nanoparticle technology against MRSA biofilms on titanium screws and coupons [13]. The considerable bacterial removal with this novel nanoparticle sonication device and improved outcomes, with a significant decrease in biofilm, when compared to the SOC treatment, can potentially have a substantial clinical implication for future PJI treatment modalities. The current mainstay for surgical PJI treatment is debridement, antibiotics, and implant retention (DAIR) procedures for acute infections (< 4 weeks), and a single- or two-stage revision for chronic infections (> 4 weeks) [18]. This approach mainly stems from concerns about biofilm formation and the ability of DAIR to eradicate infection in the presence of a matured biofilm [19]. Nevertheless, DAIR represents an appealing treatment option for both patients and surgeons due to its lower morbidity compared to other treatments [20, 21]. However, treatment failure rates of up to 50% following DAIR are concerning [22, 23]. The duration of symptoms and the time between index surgery and DAIR treatment have been previously suggested as risk factors for DAIR failure [19]. It has been previously postulated that a longer duration of symptoms may be associated with an increase in biofilm formation and maturity, which may explain why a longer duration of symptoms infers higher failure from DAIR [24]. The utilization of the novel nanoparticle sonication device can be especially helpful in these cases, as it allows for improved biofilm eradication even for fully-matured biofilm. The ultrasound energy instigates a cavitation effect within the liquid medium, leading to the formation of minute air bubbles. Following the generation of these bubbles, their rupture releases energy that propels the iron oxide nanoparticles toward the target surface, instigating an abrasive cleaning effect at a nanometer scale. This process allows for the effective cleansing of intricate and otherwise inaccessible areas which subsequently enables adequate removal of the bacteria by lavage and antibiotic treatment. Application of this novel device in DAIR procedures may offer improved bacterial eradication, potentially resulting in lower failure rates. Moreover, as previously suggested by Hameister et al. sonication treatment can help to eradicate biofilms, thus reducing the overall bioburden and potentially facilitating a single-stage over a two-stage revision, resulting in less patient morbidity and potentially lower costs [9]. Although these findings showed promising results and could potentially facilitate improved treatment for PJI, further clinical research is warranted to validate our results. Specifically, in this study, the sonication of the screws occurred after explanation (ex vivo), which limits the ability to predict the long-term success of the procedure in vivo*,* and future efficacy studies will be needed to evaluate the long-term effects of the nanoparticle sonication as a PJI treatment option.

Implementation of new technologies would often raise concerns over safety. However, the use of this novel nanoparticle sonication device demonstrated a high level of safety, as evidenced by both clinical and histological assessments of the periarticular soft tissue. These results provided strong evidence that the novel nanoparticle sonication device is well tolerated and poses no significant risks to periarticular soft tissues. The safety profile of this device is reassuring, paving the way for further research and its potential implementation as a valuable tool in the treatment of PJI. However, continued monitoring and larger-scale clinical studies will be necessary to fully establish its long-term safety and efficacy.

This study is not without its limitations. First, long-term safety-associated effects of the sonication procedure were not assessed, as the animals did not recover after the sonication process. However, histological results indicated that the sonication process did not injure the host periarticular tissue, and the nanoparticles could be removed through a simple washout step without embedding into the native tissue. Additionally, although visual inspection at the time of explanation suggested that the surrounding bone was likely colonized with bacteria, this was not confirmed by culture. In addition, while the presence of bacterial biofilms over the explanted screws was confirmed by crystal violet staining, advanced imaging technologies were not utilized to confirm its presence in the soft tissue and bone. This was an animal-model study and thus our results may not be generalizable to human subjects due to differing anatomy and pathophysiology across species. Furthermore, this animal model did not provide clinical data on the long-term efficacy of the procedure, and whether this procedure will reduce the need for further debridement and revision surgery. Additionally, animal-model studies tend to be costly in terms of time and resources and an uninfected control group was not included. Our study also did not study ultrasonication without the presence of nanoparticles. These limitations warrant further investigation in future studies to better understand the long-term safety and complete extent of bacterial colonization in the surrounding tissues.

## Conclusion

The findings of this study suggest that nanoparticle sonication technology can successfully remove attached bacterial biofilms from explanted orthopedic hardware, as well as the knee periarticular soft tissue, without damaging native periarticular tissues. The study provides initial results supporting the potential of nanoparticle sonication as a treatment option for PJI, paving the way for future clinical trials.

## Data Availability

The datasets used and/or analyzed during the current study are available from the corresponding author on reasonable request.

## References

[1] Ahmed SS, Haddad FS. Prosthetic joint infection. Bone Joint Res. 2019;8:570–2. 10.1302/2046-3758.812.BJR-2019-0340. 31832177 10.1302/2046-3758.812.BJR-2019-0340 PMC6888735

[2] Walter N, Rupp M, Hierl K, Koch M, Kerschbaum M, Worlicek M, et al. Long-Term Patient-Related Quality of Life after Knee Periprosthetic Joint Infection. J Clin Med 2021;10. 10.3390/jcm10050907. 10.3390/jcm10050907 PMC7956307 33668957

[3] Thompson O, W-Dahl A, Stefánsdóttir A. Increased short- and long-term mortality amongst patients with early periprosthetic knee joint infection. BMC Musculoskelet Disord. 2022;23:1069. 10.1186/s12891-022-06024-y. 36474195 10.1186/s12891-022-06024-y PMC9724335

[4] Premkumar A, Kolin DA, Farley KX, Wilson JM, McLawhorn AS, Cross MB, et al. Projected Economic Burden of Periprosthetic Joint Infection of the Hip and Knee in the United States. J Arthroplasty. 2021;36:1484-1489.e3. 10.1016/j.arth.2020.12.005. 33422392 10.1016/j.arth.2020.12.005

[5] Benito N, Mur I, Ribera A, Soriano A, Rodríguez-Pardo D, Sorlí L, et al. The different microbial etiology of prosthetic joint infections according to route of acquisition and time after prosthesis implantation, including the role of multidrug-resistant organisms. J Clin Med 2019;8. 10.3390/jcm8050673. 10.3390/jcm8050673 PMC6572185 31086080

[6] Josse J, Valour F, Maali Y, Diot A, Batailler C, Ferry T, et al. Interaction between staphylococcal biofilm and bone: how does the presence of biofilm promote prosthesis loosening? Front Microbiol. 2019;10:1602. 10.3389/fmicb.2019.01602. 31379772 10.3389/fmicb.2019.01602 PMC6653651

[7] Izakovicova P, Borens O, Trampuz A. Periprosthetic joint infection: current concepts and outlook. EFORT Open Rev. 2019;4:482–94. 10.1302/2058-5241.4.180092. 31423332 10.1302/2058-5241.4.180092 PMC6667982

[8] Visperas A, Santana D, Klika AK, Higuera-Rueda CA, Piuzzi NS. Current treatments for biofilm-associated periprosthetic joint infection and new potential strategies. J Orthop Res. 2022;40:1477–91. 10.1002/jor.25345. 35437846 10.1002/jor.25345 PMC9322555

[9] Hameister R, Lim CT, Lohmann CH, Wang W, Singh G. What Is the Role of Diagnostic and Therapeutic Sonication in Periprosthetic Joint Infections? J Arthroplasty. 2018;33:2575–81. 10.1016/j.arth.2018.02.077. 29599035 10.1016/j.arth.2018.02.077

[10] Josic U, Mazzitelli C, Maravic T, Fidler A, Breschi L, Mazzoni A. Biofilm in endodontics: in vitro cultivation possibilities, sonic-, ultrasonic- and laser-assisted removal techniques and evaluation of the cleaning efficacy. Polymers (Basel) 2022;14. 10.3390/polym14071334. 10.3390/polym14071334 PMC9003475 35406207

[11] Sebastian S, Malhotra R, Sreenivas V, Kapil A, Chaudhry R, Dhawan B. Sonication of orthopaedic implants: A valuable technique for diagnosis of prosthetic joint infections. J Microbiol Methods. 2018;146:51–4. 10.1016/j.mimet.2018.01.015. 29382603 10.1016/j.mimet.2018.01.015

[12] Rothenberg AC, Wilson AE, Hayes JP, O’Malley MJ, Klatt BA. Sonication of arthroplasty implants improves accuracy of periprosthetic joint infection cultures. Clin Orthop Relat Res. 2017;475:1827–36. 10.1007/s11999-017-5315-8. 28290115 10.1007/s11999-017-5315-8 PMC5449333

[13] Tran PA, O’Brien-Simpson N, Palmer JA, Bock N, Reynolds EC, Webster TJ, et al. Selenium nanoparticles as anti-infective implant coatings for trauma orthopedics against methicillin-resistant Staphylococcus aureus and epidermidis: in vitro and *in vivo* assessment. Int J Nanomedicine. 2019;14:4613–24. 10.2147/IJN.S197737. 31308651 10.2147/IJN.S197737 PMC6616172

[14] Glage S, Paret S, Winkel A, Stiesch M, Bleich A, Krauss JK, et al. A new model for biofilm formation and inflammatory tissue reaction: intraoperative infection of a cranial implant with Staphylococcus aureus in rats. Acta Neurochir (Wien). 2017;159:1747–56. 10.1007/s00701-017-3244-7. 28647798 10.1007/s00701-017-3244-7

[15] Irwin S, Wang T, Bolam SM, Alvares S, Swift S, Cornish J, et al. Rat model of recalcitrant prosthetic joint infection using biofilm inocula. J Orthop Res. 2023. 10.1002/jor.25587. 10.1002/jor.25587 37132080

[16] Fan Y, Xiao Y, Sabuhi WA, Leape CP, Gil D, Grindy S, et al. Longitudinal Model of Periprosthetic Joint Infection in the Rat. J Orthop Res. 2020;38:1101–12. 10.1002/jor.24556. 31808572 10.1002/jor.24556

[17] Harrasser N, Gorkotte J, Obermeier A, Feihl S, Straub M, Slotta-Huspenina J, et al. A new model of implant-related osteomyelitis in the metaphysis of rat tibiae. BMC Musculoskelet Disord. 2016;17:152. 10.1186/s12891-016-1005-z. 27060078 10.1186/s12891-016-1005-z PMC4826501

[18] Li C, Renz N, Trampuz A. Management of Periprosthetic Joint Infection. Hip Pelvis. 2018;30:138. 10.5371/HP.2018.30.3.138. 30202747 10.5371/hp.2018.30.3.138 PMC6123506

[19] Shohat N, Goswami K, Tan TL, Yayac M, Soriano A, Sousa R, et al. 2020 Frank Stinchfield Award: Identifying who will fail following irrigation and debridement for prosthetic joint infection: a machine learning-based validated tool. Bone Jt J. 2020;102:11–9. 10.1302/0301-620X.102B7.BJJ-2019-1628.R1/XML. 10.1302/0301-620X.102B7.BJJ-2019-1628.R1 32600194

[20] Chalmers BP, Kapadia M, Chiu Y-F, Miller AO, Henry MW, Lyman S, et al. Accuracy of predictive algorithms in total hip and knee arthroplasty acute periprosthetic joint infections treated with Debridement, Antibiotics, and Implant Retention (DAIR). J Arthroplasty. 2021;36:2558–66. 10.1016/j.arth.2021.02.039. 33750631 10.1016/j.arth.2021.02.039

[21] Boyle KK, Kapadia M, Landy DC, Henry MW, Miller AO, Westrich GH. Utilization of debridement, antibiotics, and implant retention for infection after total joint arthroplasty over a decade in the United States. J Arthroplasty. 2020;35:2210–6. 10.1016/j.arth.2020.03.029. 32279946 10.1016/j.arth.2020.03.029

[22] Kuiper JWP, Vos SJ, Saouti R, Vergroesen DA, Graat HCA, Debets-Ossenkopp YJ, et al. Prosthetic joint-associated infections treated with DAIR (debridement, antibiotics, irrigation, and retention): analysis of risk factors and local antibiotic carriers in 91 patients. Acta Orthop. 2013;84:380. 10.3109/17453674.2013.823589. 23848215 10.3109/17453674.2013.823589 PMC3768038

[23] Buller LT, Sabry FY, Easton RW, Klika AK, Barsoum WK. The preoperative prediction of success following irrigation and debridement with polyethylene exchange for hip and knee prosthetic joint infections. J Arthroplasty. 2012;27:857-64.e1-4. 10.1016/j.arth.2012.01.003. 22402229 10.1016/j.arth.2012.01.003

[24] Shao H, Li R, Deng W, Yu B, Yang D, Zhou Y, et al. Symptom duration is associated with failure of periprosthetic joint infection treated with debridement, antibiotics and implant retention. Front Surg. 2022;9:913431. 10.3389/fsurg.2022.913431. 36117805 10.3389/fsurg.2022.913431 PMC9470758

